# Spatial patterns of light‐demanding tree species in the Yangambi rainforest (Democratic Republic of Congo)

**DOI:** 10.1002/ece3.8443

**Published:** 2021-12-20

**Authors:** Nestor K. Luambua, Wannes Hubau, Kolawolé Valère Salako, Christian Amani, Bernard Bonyoma, Donatien Musepena, Mélissa Rousseau, Nils Bourland, Hippolyte S.M. Nshimba, Corneille Ewango, Hans Beeckman, Olivier J. Hardy

**Affiliations:** ^1^ Faculty of Renewable Natural Resources Management University of Kisangani Kisangani Democratic Republic of Congo; ^2^ Service of Wood Biology Royal Museum for Central Africa Tervuren Belgium; ^3^ Faculté des sciences Agronomiques Université Officielle de Mbujimayi Mbujimayi Democratic Republic of Congo; ^4^ Department of Environment Laboratory of Wood Technology Faculty of Bioscience Engineering Ghent University Ghent Belgium; ^5^ School of Geography University of Leeds Leeds UK; ^6^ Laboratoire de Biomathématiques et d’Estimations Forestières Faculty of Agronomic Sciences University of Abomey‐Calavi Cotonou Benin; ^7^ Service d'Évolution Biologique et Écologie Université Libre de Bruxelles Brussels Belgium; ^8^ Faculty of Sciences and Applied Sciences Université Officielle de Bukavu Departement de la Biologie Bukavu Democratic Republic of Congo; ^9^ Center for International Forestry Research Bogor (Barat) Indonesia; ^10^ Section de la Foresterie Institut National pour l'Etude et la Recherche Agronomique Yangambi Democratic Republic of Congo; ^11^ Resources & Synergies Development Pte Ltd Singapore Singapore; ^12^ Department of Ecology and Flora Resources Management Faculty of Sciences University of Kisangani Kisangani Democratic Republic of Congo

**Keywords:** African forest ecology, forest composition, light‐demanding species, spatial analysis, Yangambi biosphere reserve

## Abstract

Most Central African rainforests are characterized by a remarkable abundance of light‐demanding canopy species: long‐lived pioneers (LLP) and non‐pioneer light demanders (NPLD). A popular explanation is that these forests are still recovering from intense slash‐and‐burn farming activities, which abruptly ended in the 19th century. This “human disturbance” hypothesis has never been tested against spatial distribution patterns of these light demanders. Here, we focus on the 28 most abundant LLP and NPLD from 250 one‐ha plots distributed along eight parallel transects (~50 km) in the Yangambi forest. Four species of short‐lived pioneers (SLP) and a single abundant shade‐tolerant species (*Gilbertiodendron dewevrei*) were used as reference because they are known to be strongly aggregated in recently disturbed patches (SLP) or along watercourses (*G. dewevrei*). Results show that SLP species are strongly aggregated with clear spatial autocorrelation of their diameter. This confirms that they colonized the patch following a one‐time disturbance event. In contrast, LLP and NPLD species have random or weakly aggregated distribution, mostly without spatial autocorrelation of their diameter. This does not unambiguously confirm the “human disturbance” hypothesis. Alternatively, their abundance might be explained by their deciduousness, which gave them a competitive advantage during long‐term drying of the late Holocene. Additionally, a canonical correspondence analysis showed that the observed LLP and NPLD distributions are not explained by environmental variables, strongly contrasting with the results for the reference species *G. dewevrei*, which is clearly aggregated along watercourses. We conclude that the abundance of LLP and NPLD species in Yangambi cannot be unambiguously attributed to past human disturbances or environmental variables. An alternative explanation is that present‐day forest composition is a result of adaptation to late‐Holocene drying. However, results are inconclusive and additional data are needed to confirm this alternative hypothesis.

## Graphical abstract

The objective of this paper was to contribute to the elucidation of the light‐demanding species persistence puzzle in the Congo Basin rainforest canopy. Three hypotheses have been tested, which suggest: (H1) legacy of past disturbances, (H2) legacy of adaptation to a drier climate, and (H3) consequence of heterogeneity of the environment. Our results clearly support H1 for the species of short‐lived pioneer guild and do not unambiguously support a single hypothesis for the long‐lived pioneer and non‐pioneer light‐demanding guilds, although they seem to favor H2.

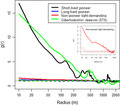

## 1 INTRODUCTION

**FIGURE 1:**
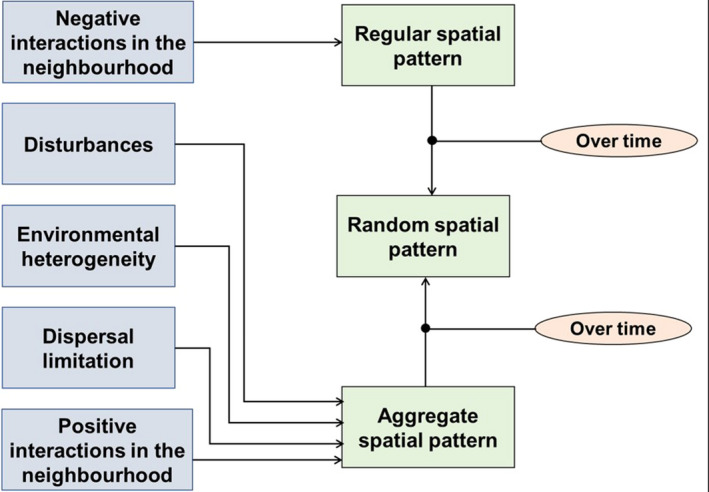
Simplified model showing the drivers of spatial pattern of trees

The spatial organization of plant species can be used to trace the imprint of past events, certain ecological processes, and mechanisms that maintain species coexistence (Fibich et al., 2016; Hardy & Sonké, 2004; Lan et al., 2012). When considering the spatial distribution of individuals of a single species, three spatial patterns are known in nature: random, regular, and aggregated (Puig, 2001; Réjou‐Méchain et al., 2011). Understanding links between these distribution patterns and their drivers is a central issue in plant population and community ecology (Fibich et al., 2016). Distribution patterns of tree species in a forest are regulated by very different factors (Figure 1). First, they are regulated by dispersion constraints (dispersal modes). Autochoric species tend to be more clustered at small spatial scales than anemochoric or zoochoric species (Kumba et al., 2013; Meunier et al., 2015). Second, biotic factors can determine spatial patterns of trees. Positive interactions (facilitation) between conspecific trees favor spatial aggregation, while negative interactions (e.g., competition, share of natural enemies) favor a regular spatial distribution (Boulangeat et al., 2012; De Araújo et al., 2014; Lortie et al., 2004). Third, distribution patterns can be regulated by resource availability so that aggregation results from environmental filtering in a heterogeneous landscape. For example, certain species aggregate in proximity to waterways or on certain soils (Condit et al., 2013; Enoki & Abe, 2004; John et al., 2007; Kearsley et al., 2017; Ripley, 1987a). Finally, disturbances create the conditions for a new succession of species, favoring the aggregation of pioneer species, a process where light availability is a key filtering factor as described hereafter.

In closed‐canopy tropical forests, light is an important resource affecting the spatial distribution of tree species. Species behave differently with respect to light availability and can be broadly divided into four regeneration guilds (Hawthorne, 1995): short‐lived pioneer species (SLP), long‐lived pioneer species (LLP), non‐pioneer light‐demanding species (NPLD), and shade‐tolerant species (STS) (Biwolé et al., 2015; Bourland et al., 2012; Meunier et al., 2015; Puig, 2001). Over the course of forest succession, which typically covers several centuries in tropical rainforests, these regeneration guilds follow one another chronologically, starting with SLP, then LLP, NPLD, and finally STS (Chazdon, 2008; Puig, 2001). Their relative abundances are therefore often used as a “forest succession clock” (Puig, 2001).

Current knowledge on the spatial distribution of tropical tree species and underlying factors are mostly based on patterns observed in the Amazon and Southeast Asia (e.g., Baraloto & Couteron, 2010; Barberis et al., 2002; John et al., 2007; Jones et al., 2006; Oliveira‐Filho et al., 1994; Potts et al., 2012; Tuomisto et al., 2003; Valencia et al., 2004; Vormisto et al., 2004). In contrast, very little information is available on the spatial patterns of species distribution in Central Africa, except a few studies centered on the effects of dispersion and habitat heterogeneity (e.g., Hardy & Sonké, 2004; Réjou‐Méchain et al., 2011).

Yet, Central African rainforests are characterized by mysteriously odd diversity and species dominance patterns: In many Central African tropical rainforests, light‐demanding species (LLP and NPLD) dominate the canopy (Bourland et al., 2015; Morin‐Rivat et al., 2017; Poorter et al., 1996; Van Gemerden et al., 2003). It has been noted that this phenomenon is less prominent in other tropical forest regions, which is one of the reasons why African tropical forests are often labeled “the odd man out” (Richards, 1952; Parmentier et al., 2007; Poorter et al., 1996). While certain studies have attempted to explain this feature through analysis of forest structure and diameter distributions, no study focused on spatial distribution patterns of these light demanders.

Therefore, the objectives of our study are to: (i) analyze the current spatial distribution of light‐demanding species by combining information on individual species, regeneration guild, and stem size and (ii) discuss the relative role of different factors determining the observed spatial pattern, by considering three hypotheses (see Table 1 and Methods).

**TABLE 1:** Main hypotheses, null models, and predictions

Hypotheses	Null models and tests	Prediction
H1—The present‐day spatial pattern of light‐demanding species or their regeneration guilds observed in the forest is a legacy of human disturbances that created large canopy gaps.	“Spatial random distribution,” tested with the pair correlation function (g) “dbh not spatially autocorrelated,” tested with Moran's index (*I*)	We would expect the light‐demanding species to be aggregated. Additionally, we would expect a positive and significant spatial autocorrelation of the dbh before the development of the local size hierarchy that is attributable to competition over time. As such, we expect rejection of the null models: *g*(*r*) > 1 and *I*(*c*) > 0
H2—The present‐day spatial pattern of light‐demanding species or their regeneration guilds observed in the forest is a legacy of adaptation to an increasingly drier climate over the last millennia because these species also have the competitive advantage of being deciduous (with deciduousness being a drought‐avoidance strategy)	“Complete spatial random distribution,” tested with the pair correlation function (*g*). “dbh not spatially autocorrelated,” tested with Moran's index (*I*)	We would expect the light‐demanding species to be deciduous. We would expect the light‐demanding species to be randomly distributed in the forest (i.e., not aggregated) and to be not spatially autocorrelated. As such, we expect acceptance of the null models: *g*(*r*) ≈ 1 and *I*(*c*) ≈ 0
H3—Abiotic filtering due to environmental heterogeneity (e.g., terrain altitude, slope, distance from waterways, and topography) explains a large portion of the variability in the spatial distribution of tree individuals considered at the species or regeneration guild level	“Species or regeneration guilds are independent of environmental variables,” tested with a canonical correspondence analysis (CCA)	Because light demanders are fast‐growing species, they should have a resource demand and therefore a preference for microhabitats. If this is the case, we would expect these species or regeneration guilds to be related to certain environmental variables

## 2 METHODS

### 2.1 Study area

**FIGURE 2:**
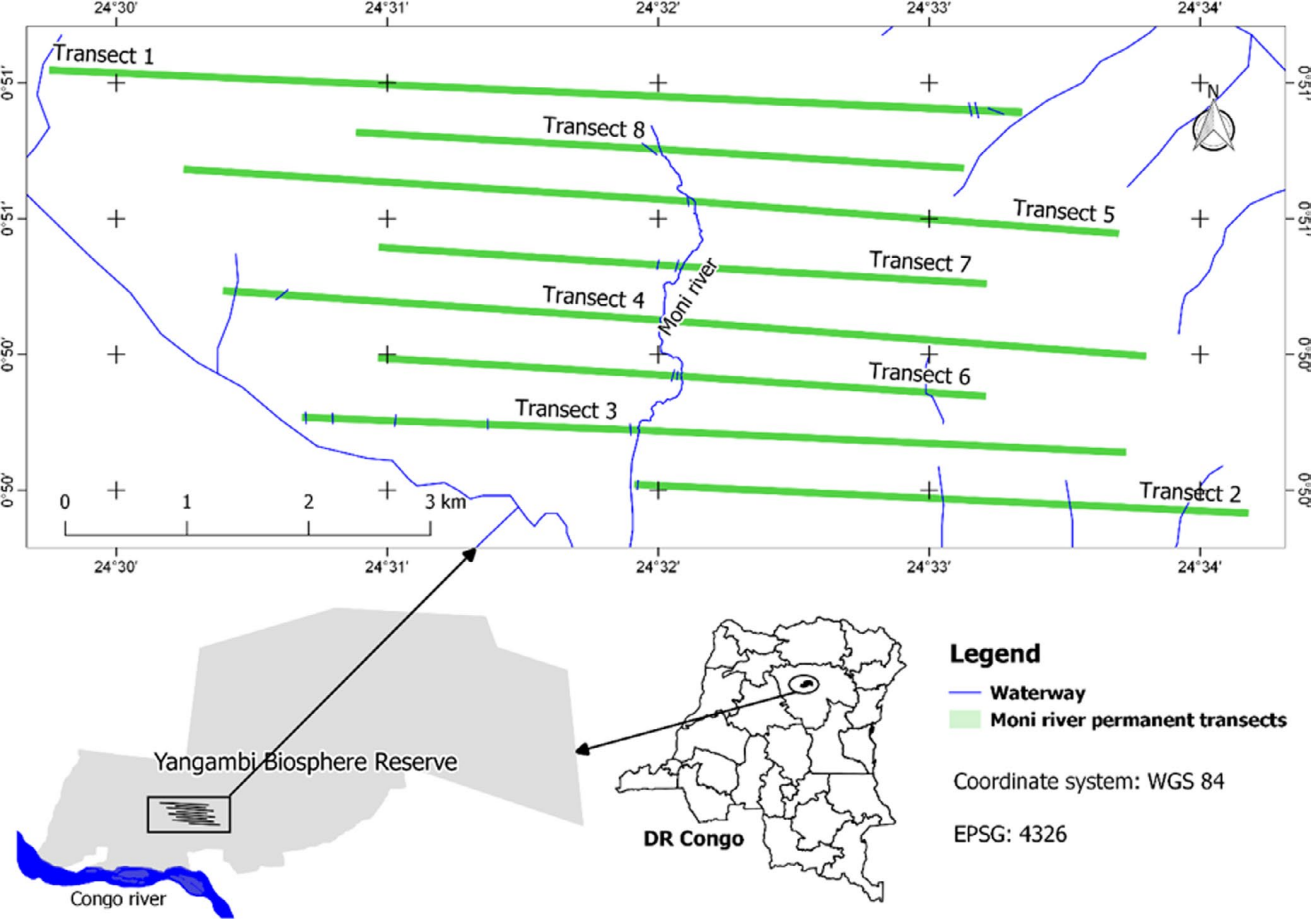
Localization of Moni River 50‐m‐wide permanent transects

The study was conducted in the Yangambi Biosphere Reserve (YBR) located in the province of Tshopo in the northeast of Democratic Republic of Congo (DRC), between 0°49′─0°51′N and 24°29′─24°35′E (Figure 2). In general, four types of terra firme forest are found in YBR: (i) young secondary forest dominated by pioneer species such as *Musanga cecropioides* R. Br. ex Tedlie and *Macaranga monandra* Müll. Arg., (ii) semi‐deciduous mixed forest dominated by long‐lived pioneer species such as *Pericopsis elata* (Harms) Meeuwen, (iii) semi‐deciduous mixed forest dominated by shade‐tolerant species such as *Scorodophloeus zenkeri* Harms, and finally (iv) evergreen monodominant forests dominated by *Gilbertiodendron dewevrei* (De Wild.) J. Léonard or *Brachystegia laurentii* (De Wild.) Louis ex Hoyle (Toirambe, 2011). The Yangambi region has the Af climate according to the Köppen classification with a slightly marked dry season (Beguin, 1958). The average annual precipitation is 1837 mm, and mean annual temperature is 25.1°C (Kombele, 2004).

### 2.2 Sampling design and data collection

The study was conducted around the Moni River where we established forest inventory plots along permanent transects oriented perpendicular to the watercourses and contours. Eight 5‐ to 8‐km‐long parallel transects, east‐west oriented and separated by ~450 m, were opened in the forest (Figure 2). These transects together cover a total length of ~50 km. Along the transects, botanical surveys were carried out in plots of 50‐m width (25 m on the two sides of the transects’ baseline) and 200 m long centered on the transect baseline. Each plot was divided into eight rectangular subplots (25 m × 50 m) to facilitate the survey. In total, 2005 subplots were established, covering a total area of 250,625 ha. This area represents a sampling rate of 10.66%.

The target species measured in the plots were the 32 of the most common light‐demanding species in YBR (Kearsley et al., 2013). Four of these species are short‐lived pioneers (SLP), 13 are long‐lived pioneers (LLP), and 15 are non‐pioneer light demanders (NPLD) (see list and details in Table S1). For comparison, we also targeted *Gilbertiodendron dewevrei*, an extremely aggregative shade‐tolerant species, for which its aggregation is probably due to a strong preference for proximity to waterways (Kearsley et al., 2017). As such, the distribution pattern of this highly aggregated species will serve as a reference to evaluate distribution patterns of the light‐demanding species.

The botanical surveys were carried out according to conventional forest inventory methods (Condit, 1998; Dallmeier, 1992; Phillips et al., 2009; Picard, 2007). In each subplot, all trees of the target species with a diameter at breast height (dbh at 1.30 m) ≥10 cm were identified, mapped (x‐ and y‐coordinates), and measured for their dbh. For trees with buttresses or any deformations at 1.30 m, measuring point was taken about 50 cm above the deformation. Trees from non‐target species were solely counted.

Data on environmental variables were recorded at subplot level. Slope and topography were observed directly in the field. The slope was measured in percentage with a Suunto clinometer (Vormisto et al., 2000). For the topography, four categories were considered: flat surface, slope, crest, and shallow. Altitude and the distance to watercourses were derived from different maps of the study area and based on the geographical coordinates of the center of each subplot.

### 2.3 Statistical analysis

Statistical analyses were carried out in R software, version R 3.6.1 (R Core Team, 2019). Analyses were performed at species level and at regeneration guild level. For the species‐level analysis, only species with a minimum of 50 individuals in the database (i.e., 15 species) were considered to ensure the robustness of the analysis, and the other species were excluded. For the guild‐level analysis, we standardized the diameter data to the mean of each species to avoid blurring the results due to interspecific differences in growth rates and maximum diameter.

Several analyses were performed. First, tree spatial pattern was assessed using tree x‐ and y‐coordinates considering a point pattern process based on the pair correlation function (PCF; Stoyan & Stoyan, 1994). This analysis was intended to measure whether tree spatial pattern is random, regular, or aggregative, and at which spatial scales. Second, Moran's *I* spatial autocorrelation index (Moran, 1950) was calculated on dbh to assess the spatial structure of tree size. This analysis allowed us to test whether cohorts of individuals of similar size established in the same geographical location, as expected after a local disturbance event (H1—human disturbance). If this occurs, we expect a positive Moran I index at short spatial distance. Finally, a canonical correspondence analysis (CCA; Ter Braak 1987) was performed to examine to what extent variables characterizing habitat heterogeneity determine the plant community structure in a multivariate framework.

#### 2.3.1 Pair correlation function (PCF)

The PCF was used to describe the spatial distribution of individuals of each species and regeneration guild. It is defined as:

(1) $$g\left(r\right)=\frac{K^{\prime }\left(r\right)}{2\pi r},r\geq 0$$

where *r* is the distance between individuals, and *K*'(*r*) is the derivative of Ripley's function *K*(*r*) (Haase, 1995; Ripley, 1987b). The univariate PCF measures the ratio of the expected density of points within a radius *r* to the total number of points per unit area (Velázquez et al., 2014). The function indicates whether the spatial pattern is random, aggregated, or regular. To this end, the deviation of the observed pattern from the null hypothesis of complete spatial random distribution (CSR) was tested by comparing the observed distribution function with the confidence envelope generated by 100 Monte Carlo simulations of the null model (Olagoke et al., 2013). When *g*(*r*) > 1 (i.e., observed distribution function above the upper limit of the confidence envelop), the spatial distribution is considered aggregated. When *g*(*r*) = 1 (i.e., observed distribution function within the confidence envelop), the spatial distribution is considered to be random, whereas *g*(*r*) < 1 (i.e., observed distribution function under the lower limit of the confidence envelop) indicates regular spatial distribution (Challis et al., 2016; Law et al., 2009). This analysis was done in the R package “spatstat” (Baddeley and Turner, 2005).

#### 2.3.2 Moran's *I* index

Moran's *I* index is a measure of spatial autocorrelation of a given attribute. Basically, this index assesses the level of similarity or dissimilarity of the concerned attribute between individuals separated by a given distance interval, *c* (Fibich et al., 2016; de Frutos et al., 2007). Moran's *I* index was calculated based on dbh. When *I*(*c*) > 0, individuals separated by a distance interval *c* are spatially autocorrelated. This means that they have more similar diameters than random individuals, whereas negative values indicate the opposite trend. Zero value suggests the absence of spatial autocorrelation, that is, substantial variation in dbh at a local scale (de Frutos et al., 2007). Results were displayed as correlogram *I*(*c*). Package “ncf” (Bjornstad and Cai, 2020) was used to calculate this index. The Mantel test was further used to test the significance of calculated Moran's index (Potts et al., 2012; Vormisto et al., 2000). The Moran index for individuals of a species or of a regeneration guild is given by:

(2) $$I\left(c\right)=\frac{N}{\sum_{i}\sum_{j\neq i}w_{\mathrm{ij}}}\times \frac{\sum_{i}\sum_{j\neq i}w_{\mathrm{ij}}\left({\text{dbh}}_{i}‐\bar{\text{dbh}})({\text{dbh}}_{j}‐\bar{\text{dbh}}\right)}{\sum_{i}{\left({\text{dbh}}_{i}‐\bar{\text{dbh}}\right)}^{2}}$$

where *N* is the total number of trees, dbh*_i_* and dbh*_j_* are the respective dbh of trees *i* and *j*, *w_ij_* = 1 if the geographical distance between *i* and *j* is included in the distance interval *c*; otherwise, *w_ij_* = 0, and $\bar{\text{dbh}}$ is the mean dbh over all trees. The distance intervals were defined as non‐overlapping ranges with upper distances equal to 100, 200, 300, up to 4000 m.

To assess whether two different species had spatially correlated dbh values, we developed a variant of Moran's *I* for intertype comparisons:

(3) $$I_{12}\left(c\right)=\frac{\sum_{i}^{N1}\sum_{j}^{N2}w_{\mathrm{ij}}\left({\text{dbh}}_{i}‐\bar{{\text{dbh}}_{1}})({\text{dbh}}_{j}‐\bar{{\text{dbh}}_{2}}\right)}{\sigma_{1}\sigma_{2}\sum_{i}^{N1}\sum_{j}^{N2}w_{\mathrm{ij}}}$$

where N1 and N2 are the total numbers of trees of, respectively, species 1 and 2, $\bar{{\text{dbh}}_{1}}$ and $\bar{{\text{dbh}}_{2}}$ are their respective mean dbh, and *σ*_1_, and *σ*_2_ are the species‐specific standard deviations of their dbh. The same distance intervals were used as for within‐species Moran's *I*.

#### 2.3.3 Canonical correspondence analysis (CCA)

Canonical correspondence analysis (CCA) is a constrained multivariate method that allows to quantify the part of the variability in the plant community structure that is actually related to environmental variables (Ter Braak, 1987; Ter Braak and Verdonschot, 1995; Parmentier et al., 2005). Here, three data matrices were prepared at the subplot level. The first (matrix 1) concerned the data of environmental variables (distance from watercourses, altitude, slope, and topography). The other two matrices contained data on tree abundance for regeneration guild (matrix 2) and for species with at least 50 individuals (matrix 3). The CCA was applied first on matrices 1 and 2, and then on matrices 1 and 3. The full model, that is, the one with all environmental variables, was constructed, and then, the most parsimonious model (with the minimum possible variables) was selected using backward elimination based on AIC. The final model was used to construct the two‐dimensional ordination plot. Permutation tests (999 simulations) were carried out to evaluate the significance of the final model, and the marginal effect of each environmental variable selected in the final model (Makarenkov and Legendre, 2002). The CCA was carried out in the package "vegan" of the R software.

### 2.4 Hypotheses

The main hypotheses, null models, and predictions are summarized in Table 1.

The first hypothesis (hereafter called “H1—human disturbance”) suggests that the present‐day dominance of light‐demanding species in African forests is a legacy of past human activity (Bourland et al., 2015; Morin‐Rivat et al., 2017; Van Gemerden et al., 2003). This hypothesis is based on the assumption that the conditions needed for recruitment of these light‐demanding canopy species do not correspond to those occurring in natural gap phase dynamics. Many of these large tree species recruit poorly in small (natural) gaps but need large‐scale clearings through disturbances such as hurricanes, river dynamics, or volcanic activity (Espírito‐Santo et al., 2014; Marra et al., 2014; Van Gemerden et al., 2003). In large parts of Central Africa, these natural disturbances are rare. Hence, an increasingly popular hypothesis is that large‐scale clearings were created by humans, for example, through slash‐and‐burn farming (Bourland et al., 2015). This farming technique gained importance throughout Central Africa during the last 1000 years (Tovar et al., 2014). Slash‐and‐burn farmlands were sufficiently large for the establishment of light‐demanding trees. Recent research suggested that since 1885, slash‐and‐burn activities declined substantially, both in intensity and in geographical extent, because colonial administrations concentrated people and villages along primary communication axes (Morin‐Rivat et al., 2017). Therefore, many forest areas in Central Africa were “abandoned” and the former farmland patches were left to forest succession. As such, former farmland patches would today be forests of 100–200 years old, which corresponds to the last stage of forest succession, when long‐lived, light‐demanding pioneer trees are being replaced by shade‐tolerant species (Chazdon, 2008).

The second hypothesis (hereafter called “H2—drought adaptation”) suggests that the present‐day dominance of light‐demanding species in Central African forests is a legacy of adaptation to a drier climate (Parmentier et al., 2007). Central African tropical forests mostly receive <2000 mm yr^−1^, which is substantially drier than Asian and South American forests (Philippon et al., 2019). Therefore, they are more dominated by deciduous species (Parmentier et al., 2007) because deciduousness is a drought‐avoidance strategy (Enquist and Enquist, 2011; Fauset et al., 2012; Vico et al., 2017). Furthermore, deciduousness is often associated with fast‐growing light‐demanding canopy specialists because larger trees are more vulnerable to drought stress (Bennett et al., 2015; Hubau et al., 2019). African forests experienced a long‐term drying trend since the mid‐Holocene (since ~5000 years BP) as attested in marine and freshwater records for West Africa (Weldeab et al., 2007), the Congo Basin (Schefuß et al., 2005), and East Africa (Russell and Johnson, 2005). Therefore, the present‐day abundance of light‐demanding trees in Central Africa might be explained by their deciduousness, which gave them a competitive advantage during late‐Holocene long‐term drying.

The third hypothesis (hereafter called “H3—environmental filtering”) suggests that abiotic filtering due to environmental heterogeneity (e.g., terrain altitude, slope, distance from waterways, and topography) explains a large portion of the variability in the spatial distribution of certain tree species or perhaps entire regeneration guilds (Hardy & Sonké, 2004; Réjou‐Méchain et al., 2011). Because light‐demanding species are fast‐growing species, they have large resource demands. Therefore, they might prefer microhabitats that are rich in resources such as water, soil type, or nutrients.

### 2.5 Predictions

If H1 (human disturbance) is true, past slash‐and‐burn activities would have created a patchwork of regenerating forests causing an aggregated distribution of light‐demanding species. We would then expect a pair correlation function that rejects the null model that the spatial distribution is completely random (*g*(*r*) > 1 for small *r*; Table 1), in particular for the SLP guild if the disturbance is recent and the LLP (and possibly the NPLD) guild if the disturbance is ancient. Furthermore, we would expect Moran's index that rejects the null hypothesis that the dbh distribution is not spatially autocorrelated (*I*(*c*) > 0) because trees that settled at the same time in a gap created by a disturbance should have more or less similar diameters.

If H2 (drought adaptation) is true, the abundance of light‐demanding species would be a legacy of drought adaptation rather than recurrent disturbances. If no other processes favor their aggregation (e.g., habitat filtering, limited dispersal), we would expect a pair correlation function showing a completely random spatial distribution (*g*(*r*) ≈ 1; Table 1). Furthermore, we would expect Moran's index that retains the null hypothesis that the dbh distribution is not spatially autocorrelated (*I*(*c*) ≈ 0). We expect this because if the species composition is a legacy of drought adaptation, this would have happened over a very long‐time window (thousands of years), giving ample time for the dbh distribution to diversify.

If H3 (environmental filtering) is true for a certain species or guild, we would expect a strong relation with a certain environmental variable in the canonical correspondence analysis (CCA). This would reject the null hypothesis that species or regeneration guilds are independent of environmental variables. In addition, we would expect an aggregated pattern (*g*(*r*) > 1) but no spatial autocorrelation (*I*(*c*) ≈ 0) because individuals of these species would aggregate in areas with favorable environmental conditions, and have done so for relatively long‐time windows, allowing the dbh distribution to diversify.

## 3 RESULTS

### 3.1 Overview of the data

A total of 84678 trees were registered in all transects. Our target species represented 8.88% of this total. The SLP guild had 834 trees (3.33 trees ha^−1^), LLP 800 trees (3.19 trees ha^−1^), NPLD 4694 trees (18.73 trees ha^−1^), and *G*. *dewevrei* 1195 trees (4.77 trees ha^−1^). More details and descriptive statistics of dbh for these species are presented in Table S2.

### 3.2 Spatial pattern of species and regeneration guilds

**FIGURE 3:**
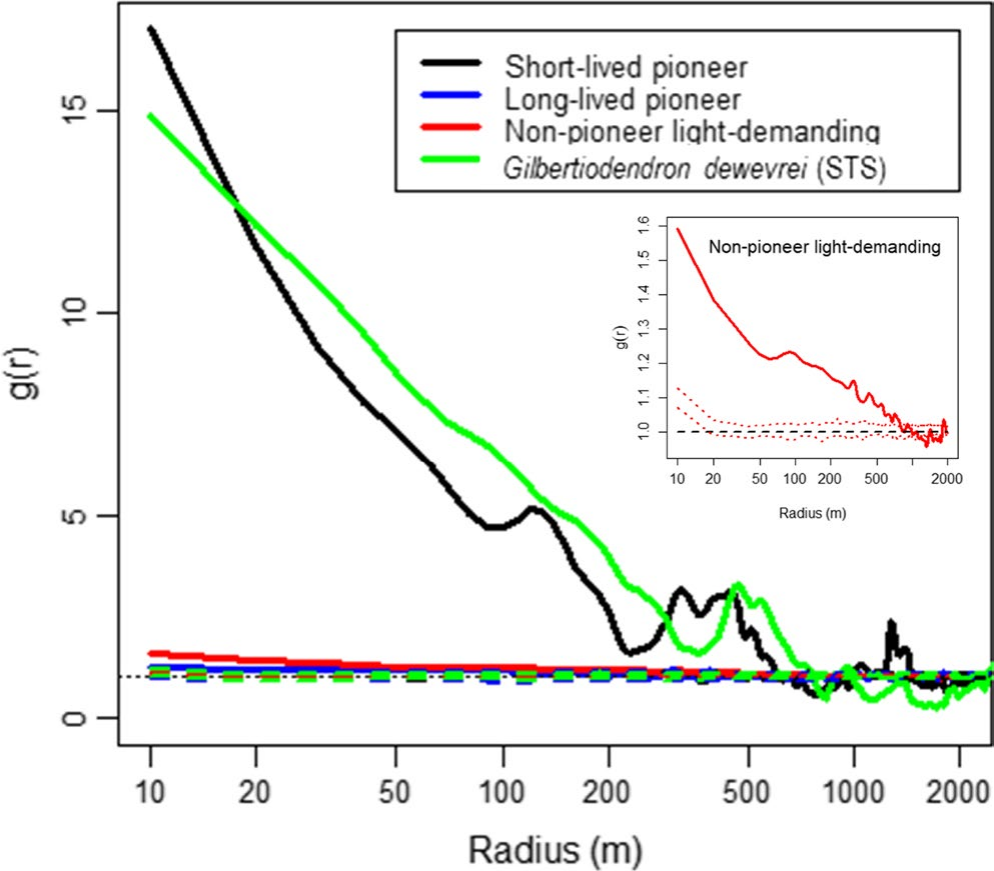
Pair correlation function *g*(*r*) of individuals from each regeneration guild. The low aggregation of the NPLD guild has been highlighted as an inset where the scale of the vertical axis was readjusted

Spatial pattern analysis at the guild level revealed a particularly strong aggregated pattern (*g* > 1 at short distances) for the SLP and STS (*G*. *dewevrei*) guilds, extending up to 590 m for the SLP and 760 m for the STS (Figure 3). For these guilds, the density of trees at 10 m of existing trees was 15–17 times higher than the average tree density (*g*_(_ *_r _*_= 10 m)_ ≈ 15 to 17, Figure 3). Trees of the NPLD guild also formed aggregates up to 800 m but with rather low *g*(*r*) values (*g*_(_ *_r _*_= 10 m)_ ≈ 1.6, Figure 3) as compared to SLP and STS. Trees of the LLP guild had *g*(*r*) values close to 1 and generally did not form aggregates. If they did form aggregates, the radius was very small, ranging from 20 to 120 m (Figure 3).

**FIGURE 4:**
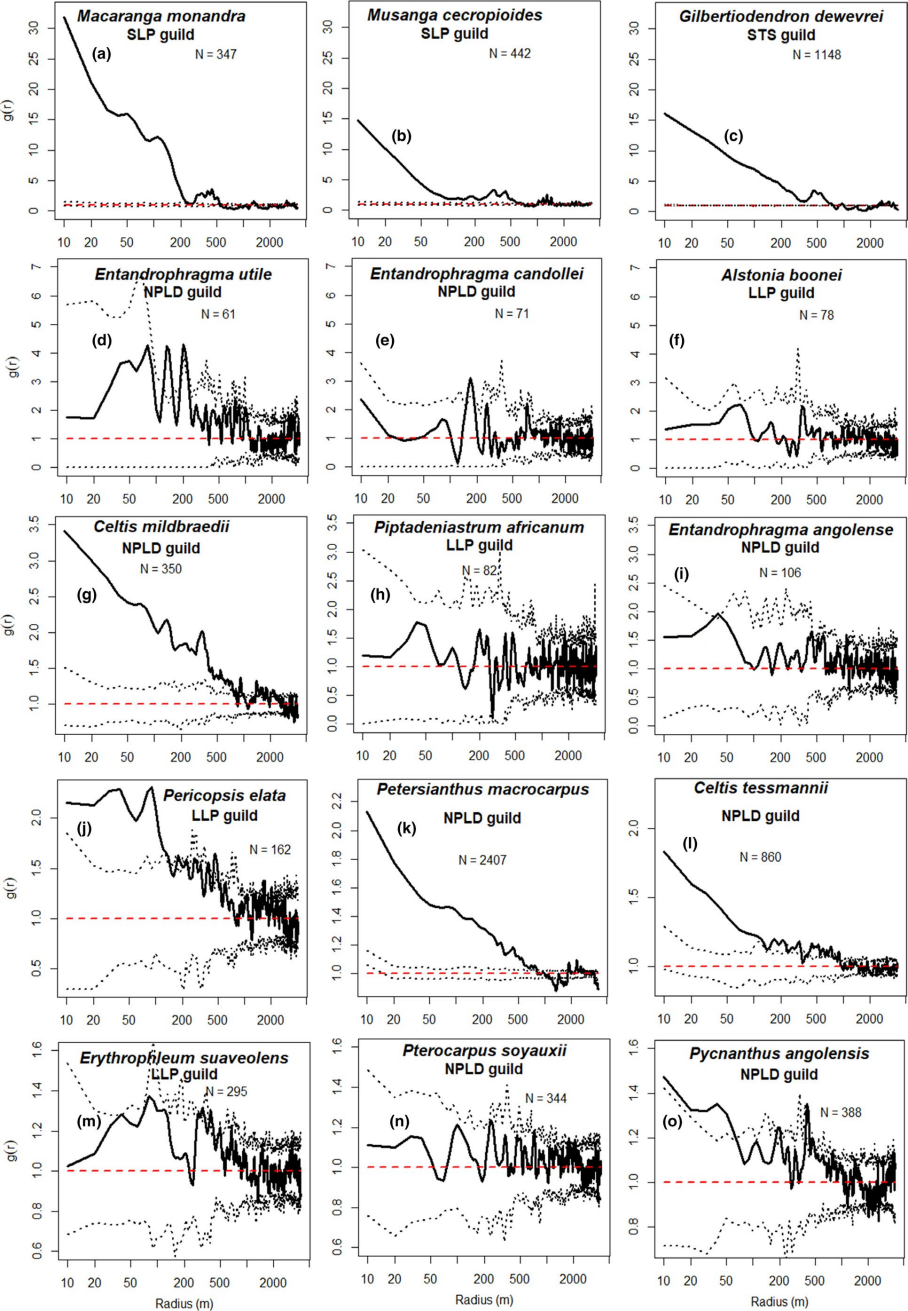
Intraspecific pair correlation function *g*(*r*) of individuals from different species. *N* refers to the sample size available. The dotted lines delimit the 95% interval expected under a random distribution of trees. Note that *y*‐axis scales gradually diminish from top to bottom, with the top row containing the largest amplitude (*g*(*r*) between 0 and 30) and the bottom row containing the narrowest amplitude (*g*(*r*) between 0 and 1.6)

At the species level, we noted great differences. Within the SLP species, *M*. *cecropioides* and *M*. *monandra* display strongly aggregated spatial distribution (*g*(*r*) up to 30) up to 660 and 230 m (Figure 4b,a). Most of the species in the LLP guild (Figure 4f,h,m) had random distribution (*g*(*r*) ≈ 1) except *P*. *elata*, which had a moderately aggregated distribution (*g*(*r*) up to 2.2), but only up to 140 m (Figure 4j). The NPLD guild is dominated by species showing random distribution except *P*. *macrocarpus*, *C*. *tessmannii*, *C*. *mildbraedii*, and *P*. *angolensis*, which had moderately aggregated distribution (*g*(*r*) up to 3.5) up to 810, 590, 740, and 60 m, respectively. *G*. *dewevrei* had a strongly aggregated distribution (*g*(*r*) up to 17) up to 760 m (Figure 4c). It should be noted that the sample size strongly conditioned the width of the confidence envelopes around the *g*(*r*) functions (Figure 4) and hence the power to detect significant aggregation pattern. For example, it is possible that *Entandrophragma utile* (*N* = 61) is actually more aggregated than *Petersianthus macrocarpus* (*N* = 2407) in the sense that its *g*(*r*) function tends to be higher at short distances (*r* < 500 m), but the *g*(*r*) function was clearly outside of the confidence envelope only for the second species (Figure 4c,k).

### 3.3 Spatial autocorrelation of DBH

**FIGURE 5:**
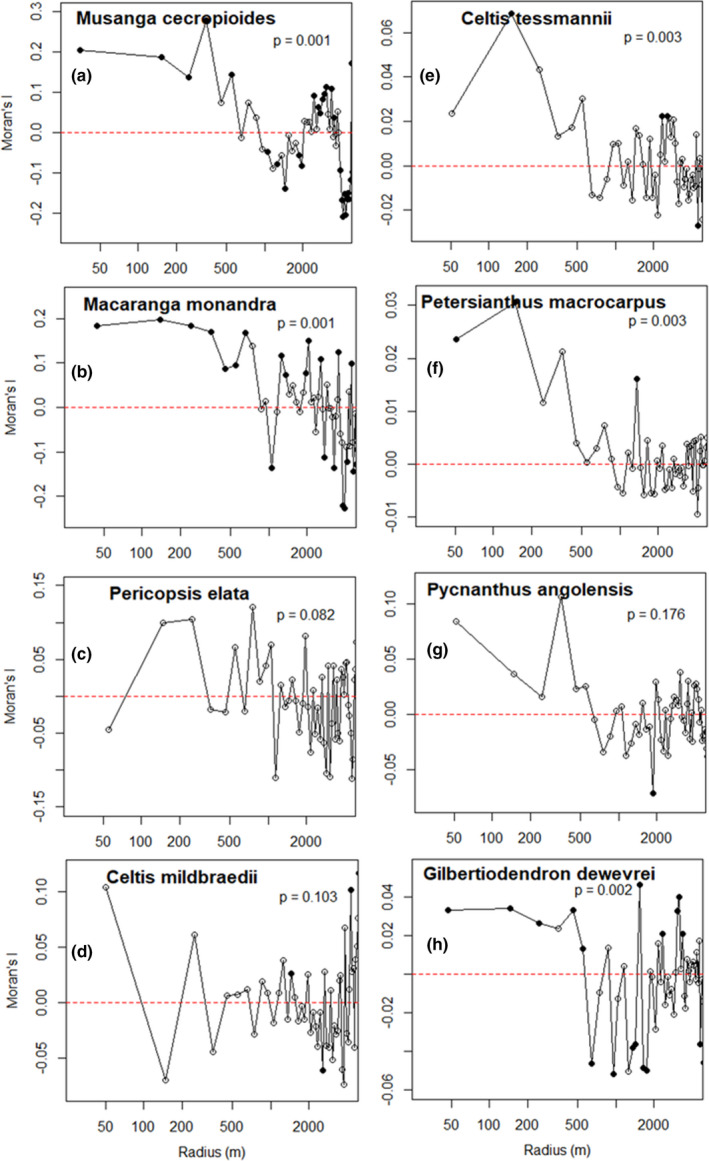
Spatial autocorrelograms (Moran's *I*) for dbh of the species that are strongly or moderately aggregated (Figure 4). Filled symbols indicate values significantly departing from the 95% confidence envelopes, contrary to open symbols. The *p*‐value refers to a Mantel test between the matrices of *I_ij_* values and ln(*d_ij_*) values

**FIGURE 6:**
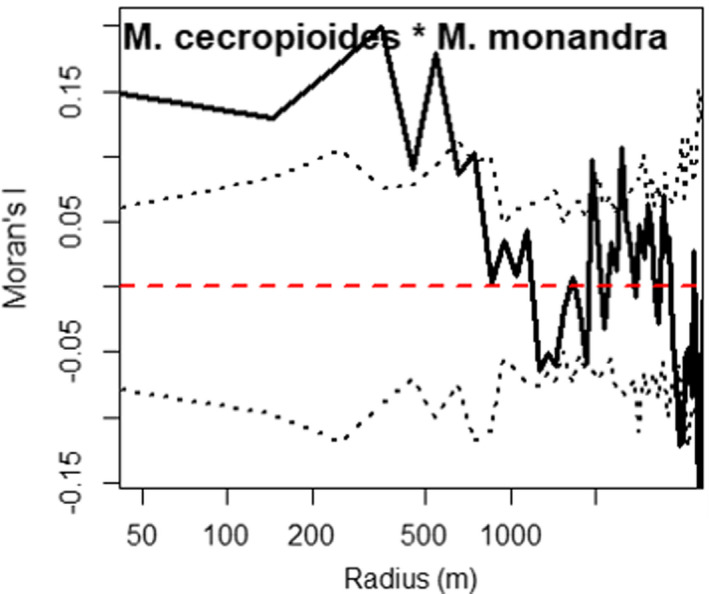
Spatial correlation of the dbh between SLP species pairs. Stippled lines delimit the 95% confidence envelopes under the null hypothesis that dbh are not spatially correlated between species

Species that were not significantly aggregated did not show any spatial autocorrelation of their dbh. For the eight species with high or moderately aggregated distribution (see Section 3.2), Moran's *I* values show that only five had a positive and significant spatial autocorrelation of dbh (Figure 5), indicating that spatially close individuals have similar dbh in these species. The autocorrelation extended up to 250 m for *G*. *dewevrei*, 450 m for *M*. *cecropioides*, 650 m for *M*. *monandra*, 250 m for *C*. *tessmannii*, and between 150 and 250 m for *P*. *macrocarpus* (Figure 5). However, *I*(*c*) for *G*. *dewevrei* ranges only up to 0.04, while for the SLP, it is up to 0.2. Only *P*. *angolensis* and *C*. *mildbraedii* reach *I*(*c*) values comparable to the SLPs. No spatial correlation of dbh was observed between species (Figure S1), except between the two most abundant SLP species, *M*. *cecropioides* and *M*. *monandra*, showing significant positive spatial autocorrelation up to 650 m (Figure 6). This indicates that spatially close individuals of these two species often have dbh deviating in the same direction from the respective species means.

### 3.4 Influence of environmental variables on local species assemblages

Results of the CCA revealed that the altitude and the distance from watercourses were the main explanatory variables in the ordination at the species level, whereas the slope and topography in addition to altitude and the distance from watercourses were important for the ordination of species’ guilds (Table 2). Environmental variables explain 23.73% of the variation in the ordination of guilds, and altitude was by far the most important variable (93.97% of the variance explained by environmental variables). Regarding species, environmental variables explain only 8.47% of the total variation in the ordination of species, and altitude was also the most important (90.25% of the variance explained by environmental variables) (Table 2).

**TABLE 2:** Comparison of different CCA models to explain the location of trees according to species or regeneration guild in relation to environmental variables

	*df*	Chi‐square	*F*	Pr(>*F*)	Proportion explained (%)
Regeneration guild including *G. dewevrei* (constrained ordination = 23.73%)					
Model: Abundance ~ Slope + Distance.from.wetland + Altitude + Topography (*df* = 6, *F* = 94.89, *p* = .001)					
Canonical axes					
CCA1	1	0.410	562.26	0.001	98.59
CCA2	1	0.005	7.66	0.024	1.34
Environmental variables					
Slope	1	0.002	2.84	0.040	1.64
Distance from wetland	1	0.002	3.42	0.031	1.98
Altitude	1	0.118	162.38	0.001	93.97
Topography	3	0.009	4.16	0.001	2.41
Regeneration guild excluding *G*. *dewevrei* (constrained ordination = 7.7%)					
Canonical axes					
CCA1	1	0.071	145.38	0.001	99.92
CCA2	1	0.00006	0.121	0.874	0.08
Environmental variables					
Slope	1	0.002	4.13	0.026	3.10
Altitude	1	0.067	136.46	0.001	96.9
Species including *G. dewevrei* (constrained ordination = 8.47%)					
Model: Abundance ~ Distance.from.wetland + Altitude (*df* = 2, *F* = 84.15, *p* = .001)					
Canonical axes					
CCA1	1	0.44	162.91	0.001	96.80
CCA2	1	0.01	5.39	0.001	3.20
Environmental variables					
Distance from wetland	1	0.016	5.73	0.001	9.75
Altitude	1	0.145	53.05	0.001	90.25
Species excluding *G. dewevrei* (Constrained ordination = 2.1%)					
Canonical axes					
CCA1	1	0.08	30.72	0.001	81.56
CCA2	1	0.015	5.56	0.001	14.76
Environmental variables					
Slope	1	0.007	2.67	0.002	13.80
Distance from wetland	1	0.013	4.94	0.001	25.70
Altitude	1	0.031	11.64	0.001	60.50

**FIGURE 7:**
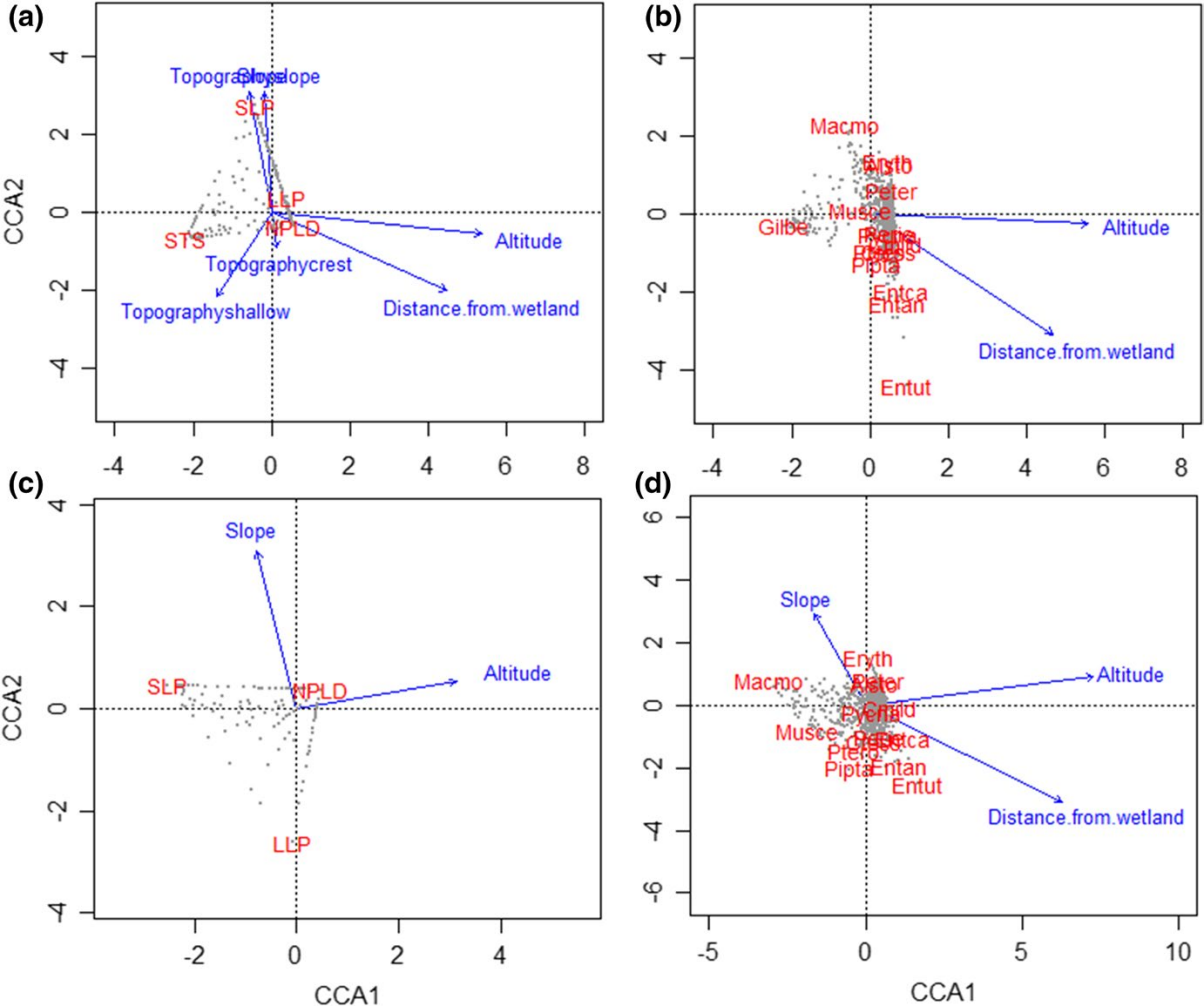
Distribution of species and their regeneration guilds in the space defined by the first two axes derived in canonical correspondence analysis (CCA). (a) CCA ordination diagram of regeneration guilds. (b) CCA ordination diagram of species. (c) CCA ordination diagram of regeneration guilds without G. dewevrei. (d) CCA ordination diagram of species without G. dewevrei. Environmental variables are indicated by vectors; vector length indicates the relative weight of a given variable in the ordination, and the direction represented by the arrow indicates the correlation of that variable with each axis. The means of the environmental variables are at the origin (0.0); values above the mean of a given variable lie along its corresponding vector in the direction of the arrow, and values below the mean lie along the extension of the vector in the opposite direction. The species names are abbreviated as follows: Macmo = Macaranga monandra, Musce = Musanga cecropioides, Alsto = Alstonia boonei, Eryth = Erythrophleum suaveolens, Perie = Pericopsis elata, Pipta = Piptadeniastrum africanum, Cmild = Celtis mildbraedii, Ctess = Celtis tessmannii, Entan = Entandrophragma angolense, Entca = Entandrophragma candollei, Entut = Entandrophragma utile, Peter = Petersianthus macrocarpus, Ptero = Pterocarpus soyauxii, Pycna = Pycnanthus angolensis, and Gilbe = Gilbertiodendron dewevrei. The regeneration guild names are abbreviated as follows: SLP = short‐lived pioneer, LLP = long‐lived pioneer, NPLD = non‐pioneer light‐demander, and STS = shade‐tolerant species. The sites are represented by gray dots

The CCA showed that the regeneration guilds were grouped into three categories: (i) the *G*. *dewevrei* (STS) was located in low altitude environment, close to watercourses and characterized by shallows; (ii) the LLP and NPLD guilds were located in the upland environment and high altitude; and (iii) the SLP guild was linked to a sloping environment (Figure 7a). The ordination of species revealed two main groups: the group of *G*. *dewevrei* that is located in low altitude environment and close to watercourses; and the group of LLP and NPLD guilds species that is located in the upland environment and high altitude (Figure 7b). However, it should be noted that most LLP and NPLD species or LLP and NPLD guilds are close to the biplot origin.

Removal of *G*. *dewevrei* from both analyses and keeping only the light‐demanding species showed strong changes in the CCA results (Figure 7c,d). Slope, altitude, and distance from watercourses are now the main explanatory variables for species ordination, whereas only the first two are now the main explanatory variables for the ordination of regeneration guilds. Environmental variables now explain only 2.1% of the variation in species ordination and 7.7% for regeneration of guilds. In both ordinations, altitude remains the most important variable (Table 2).

## 4 DISCUSSION

Our study heavily draws on forest succession theory (also called sylvigenetic cycle theory). Hallé et al. (1978) explained that when the canopy opens following a disturbance, several phases of plant succession characterized by a turnover of the dominant regeneration guilds follow one after the other before returning to the initial state of the forest. The canopy opening allows the entry of a large amount of light, which is a determining factor for the colonization and establishment of light‐demanding species (Delcamp et al., 2008; Marra et al., 2014; Puig, 2001). Based on this theory, a canopy opening first favors the establishment of a large number of individuals of strongly regenerating short‐lived pioneer species (i.e., the SLP guild), which are relatively quickly (i.e., after a few decades; Chazdon, 2008) replaced by long‐lived pioneers (LLP), then by non‐pioneer light demanders (NPLD) and eventually, after hundreds of years, by shade‐tolerant species (STS). This theory is applicable both on large‐scale openings and on small‐scale openings. Large‐scale openings typically occur in anthropogenic landscapes, where slash‐and‐burn activities create large gaps that are subsequently left to forest succession (Morin‐Rivat et al., 2017; Van Gemerden et al., 2003). Small‐scale openings typically occur in old‐growth tropical forests, where species composition is determined by natural gap phase dynamics. Both types of canopy openings have occurred frequently in the African rainforest, but the question is which of these has left a significant imprint on present‐day species composition?

Below, we discuss each of our hypotheses in light of the results obtained by our analysis. The first (H1—human disturbance) draws on a dominant influence of large‐scale openings by human activities, which may have changed forest patches over relatively short timescales (hundreds of years). The second (H2—drought adaptation) draws on a dominant influence of small‐scale openings and natural gap phase dynamics, which may have changed species composition of large parts of the African rainforest over relatively long timescales (thousands of years). Finally, we discuss other factors that may influence spatial distribution such as environmental filtering (H3) or dispersal mode.

### 4.1 H1—human disturbance hypothesis

Hypothesis 1 is based on the logic that after slash‐and‐burn activities, SLP species regenerate abundantly and locally in patches so that they show spatial aggregation plus a cohort effect with positive and significant autocorrelation of dbh. The aggregated distribution and autocorrelation of dbh is expected to be more or less maintained over relatively short periods (i.e., 100–200 years). Our results show that this works well for the SLP regeneration guild (*M*. *cecropioides* and *Macaranga* spp.). SLP species had a much higher degree of aggregation and spatial autocorrelation than LLP, NPLD, and even STS (*G*. *dewevrei*). High spatial autocorrelation of the dbh of SLP species indicates a synchronization of their establishment across time in areas that have undergone recent disturbance, that is, in the past few decades. However, in this study, we test whether this hypothesis also works for old disturbances (i.e., >100 years ago), by focusing on the LLP (or even NPLD) guild. Apart from *P*. *macrocarpus* and *C*. *tessmannii*, species in these guilds showed no or little aggregation or spatial autocorrelation of their dbh, suggesting that they are randomly distributed throughout the forest. Here, we discuss three possible scenarios that may explain this.

A first scenario is that the LLP and NPLD abundance in the canopy is indeed a legacy of past slash‐and‐burn activities, as H1 suggests, but that aggregation and spatial autocorrelation heavily decreased over time due to competition between neighboring tree individuals (Fibich et al., 2016; Suzuki et al., 2008). In this case, tree mortality, recruitment, and growth heterogeneity over time eventually erased traces of initial aggregation and spatial structure of dbh. In Asian forests, it has indeed been illustrated that the degree of aggregation decreases with competitive processes (Fibich et al., 2016). On a given site, the spatial distribution pattern is not static, but changes according to the plant succession stage (Felinks & Wiegand, 2008; Greig‐Smith, 1952; Malkinson & Kadmon, 2007; Suzuki et al., 2008; Velázquez et al., 2014). Yet, an argument against this scenario is that it seems unlikely that aggregation and spatial autocorrelation almost entirely disappeared in the course of just more than 100 years.

A second scenario is that the LLP and NPLD abundance in the present‐day canopy is indeed a legacy of past slash‐and‐burn activities, but these activities were so intense and frequent that they affected the whole forest, rather than scattered patches. Recurring disturbance in previously disturbed forest creates spatial heterogeneity and blurs aggregated patterns. Yet, an argument against this scenario is that population numbers in Central Africa in the centuries preceding the colonial era were relatively low due to the Transatlantic slave trade (Lovejoy, 1989). This does not favor the scenario of intense, recurring disturbance.

A third scenario is that the LLP and NPLD abundance in the present‐day canopy is not a legacy of past slash‐and‐burn activities and that other factors are at stake (i.e., H2). We conclude that our results do not confirm H1 (human disturbance), but they also do not unambiguously falsify it, because traces of human activity patterns may have been erased over time.

### 4.2 H2—drought adaptation hypothesis

If not ancient disturbances, what else could explain the present‐day abundance of light‐demanding species? Forest ecosystems are known to be resilient. When an ecosystem is faced with the constraints of a changing external environment, it evolves toward the most resistant state (Cropp and Gabric, 2002). African rainforests are much drier than, for example, South American rainforests (Parmentier et al., 2007). As such, we expect a higher abundance of drought‐adapted species in African rainforests. Ignoring environmental heterogeneity, we would expect that these species are randomly distributed throughout the African rainforest, as these relatively dry conditions occur everywhere. Our results seem to support this hypothesis. All of the LLP and NPLD species in our dataset are either deciduous or semi‐deciduous except one (*Pycnantus angolensis*). In addition, most of the LLP and NPLD species are randomly distributed in the study site. Based on this combination of patterns, H2 could explain the presence and abundance of these species in the study site given that deciduousness is known as a drought‐avoidance strategy (Condit et al., 2000; Fauset et al., 2012; Vico et al., 2017). The forest would have responded to increasingly drier conditions over the last millennia by multiplying the number of individuals of deciduous or semi‐deciduous species to the detriment of evergreen species. This scenario is supported by the fact that such shifts toward more deciduous individuals in the canopy are even observed during the last decades due to multidecadal drying trends in West Africa (Aguirre‐Gutiérrez et al., 2020; Fauset et al., 2012) and in the Congo Basin (Zhou et al., 2014). As such, we argue that their deciduousness is perhaps more important to explain the dominance of light demanders in the African rainforest than the fact that they are light‐demanding. Yet, the second hypothesis cannot be unambiguously accepted, considered that five out of 28 LLP and NPLD species are (weakly) aggregated. Nevertheless, H2 is a promising hypothesis for further research. Particularly, paleobotanical research such as charcoal analysis could provide valuable insights to test this hypothesis (Hubau et al., 2015).

### 4.3 H3—habitat heterogeneity hypothesis

The effect of habitat heterogeneity on spatial distribution was tested by the CCA. The results of the CCA showed overall that the inventoried species had affinities with certain environmental variables such as altitude and distance from watercourses. These affinities decreased significantly when *G*. *dewevrei* was removed from the analysis. Environmental variables then explained less than 5% of the spatial variation's abundance of light‐demanding species and about 10% of that of entire LLP and NPLD regeneration guilds. Unexpectedly, the habitat heterogeneity does not locally influence the abundance and spatial organization of light‐demanding species in the site (Getzin et al., 2008; Wiegand et al., 2007). On the contrary, the fact that the addition of *G*. *dewevrei* improves the share of variance explained by habitat heterogeneity shows some effect of this factor on its abundance. This is supported by the fact that individuals of this species are very often located near watercourses and thus low relative altitude in the landscape (Kearsley et al., 2017). The observed low autocorrelation's dbh of this species may have a different explanation than that of light‐demanding species. Indeed, individuals of this species interact to completely close the canopy and form a very thick litter so as to favor its regeneration to the detriment of those of other species (Kearsley et al., 2017; Torti et al., 2001). Thus, several seeds scattered at the same time around the mother tree can develop together with more or less the same dbh. Consequently, habitat heterogeneity might be here, more determinant for the abundance and spatial organization of *G*. *dewevrei* than light‐demanding species.

### 4.4 Alternative explanation

There are a few factors other than human disturbance, drought adaptation, or environmental filtering. In general, the dispersal mode of diaspores plays an important role in the spatial organization of individual's forest species. It reflects the capacity of species to disperse their diaspores in space. The dispersal distance of diaspores is linked to the agents of dissemination (Kumba et al., 2013; Puig, 2001). The dispersed diaspores, while awaiting favorable conditions for the dormancy breaking (Deval, 1967), first feed the soil seed bank. Fibich et al. (2016) attributed the aggregation of pioneers after cultivation to the dispersal factor. It is true that not all diaspores are dormant. Although they may begin to grow, their development is further determined by conditions favorable to the growth of the species. For most of the light‐demanding species that we inventoried, their dispersal is mainly ensured by animals and the wind (Meunier et al., 2015). These modes of dispersal allow the dispersal of diaspores over large distances and without any particular spatial organization, which could favor an aggregated pattern of individuals. Thus, the role of the dispersal mode seems subsidiary in the case of the spatial organization of light‐demanding individuals. On the contrary, the diaspores of species with a low dispersal capacity are limited around mother plants, which a priori leads to the formation of aggregates (Kumba et al., 2013). This could justify the aggregation of *G*. *dewevrei* whose seeds are dispersed by autochory.

### 4.5 Limitation and future research

This study could not clearly identify the major factor of spatial organization of light‐demanding species individuals. Our analysis of habitat heterogeneity was based on only a few variables including altitude, slope, and distance from watercourses. These variables are not the only ones that define the environment. Other variables such as soil properties could have provided additional insights in our results. However, it should be noted that there is a significant correlation between soil properties and, for example, altitude (Charan et al., 2013; He et al., 2016; Saeed et al., 2014). Moreover, the signal of past disturbances could be identified by dendrochronological and anthracological data. This can be used to discriminate between the human disturbance hypothesis (H1) and the drought adaptation hypothesis (H2). Furthermore, expanding the inventory to include more shade‐tolerant species could shed light on differences in spatial patterns. Also, including evergreen species in the inventory would allow to test whether deciduous LLP and NPLD species differ from evergreen LLP and NPLD species in terms of spatial aggregation, which would allow to better test H2.

## 5 CONCLUSIONS

The aim of this study was to analyze the present‐day spatial distribution model of light‐demanding species in the Yangambi Biosphere Reserve (YBR) and to discuss the role of each factor in this spatial distribution. The pair correlation function revealed a high degree of aggregation and autocorrelation of dbh for SLP guild tree individuals. This spatial distribution has been associated with disturbances over the last decade. A difference in the distribution pattern was observed between LLP or NPLD guild species. The species spatial distribution and relationship are more dependent on the propagation characteristics and the mode of seed dispersal, reflecting the population regeneration processes, the interspecific and intraspecific competition level. No major factors were found for the spatial organization of these species. Our spatial approach therefore does not allow to unambiguously confirm or exclude the first two hypotheses, although our results seem to favor H2 (drought adaptation) because most species in the dataset seem to be randomly distributed in the forest, especially the LLP species. Concerning *G*. *dewevrei*, its individuals preferred environments near watercourses and low altitudes, and its mode of dispersal (autochory) favors to aggregation. Limited seed dispersal and habitat heterogeneity therefore would be major factors in its aggregation. Based on the comparison between the affinity degree of light‐demanding species and the one of *G*. *dewevrei* with the environmental variables, we would exclude the third hypothesis for the light‐demanding species. Finally, we think that further insights require a multidisciplinary approach combining data of soil properties, tree dating, anthracology, history, and repeated forest inventories to elucidate the major factors determining the spatial distribution of light‐demanding species in our study site.

## CONFLICTS OF INTEREST

The authors declare no conflict of interest.

## AUTHOR CONTRIBUTIONS

**Nestor K. Luambua:** Data curation (lead); Formal analysis (equal); Funding acquisition (equal); Investigation (lead); Methodology (equal); Project administration (equal); Writing – original draft (equal); Writing – review & editing (equal). **Wannes Hubau:** Conceptualization (equal); Methodology (equal); Writing – original draft (equal); Writing – review & editing (equal). **Kolawolé Valère Salako:** Formal analysis (equal); Methodology (equal); Writing – original draft (equal); Writing – review & editing (equal). **Christian Amani:** Writing – review & editing (equal). **Bernard Bonyoma:** Investigation (supporting); Writing – review & editing (equal). **Donatien Musepena:** Investigation (supporting); Writing – review & editing (equal). **Mélissa Rousseau:** Conceptualization (equal); Data curation (equal); Methodology (equal); Project administration (equal); Writing – review & editing (equal). **Nils Bourland:** Conceptualization (equal); Data curation (equal); Funding acquisition (supporting); Investigation (supporting); Methodology (supporting); Project administration (supporting); Writing – original draft (supporting); Writing – review & editing (supporting). **Hippolyte Nshimba:** Conceptualization (equal); Methodology (equal); Project administration (equal); Writing – original draft (supporting). **Corneille Ewango:** Formal analysis (supporting); Methodology (equal); Writing – original draft (supporting). **Hans Beeckman:** Conceptualization (lead); Funding acquisition (lead); Methodology (equal); Project administration (lead); Writing – review & editing (equal). **Olivier J. Hardy:** Formal analysis (lead); Methodology (equal); Software (lead); Supervision (lead); Validation (lead); Writing – original draft (equal); Writing – review & editing (equal).

## Supporting information

Figure S1

Table S1‐S2

## Data Availability

The data collected and analyzed for this paper are available on Dryad: https://doi.org/10.5061/dryad.qv9s4mwdf.
