# Supplementary figures and images for: Spatial patterns of light‐demanding tree species in the Yangambi rainforest (Democratic Republic of Congo)
Source: Ecol Evol. 2021 Dec 20;11(24):18691–707. doi: 10.1002/ece3.8443 (PMC8717288; doi:10.1002/ece3.8443)

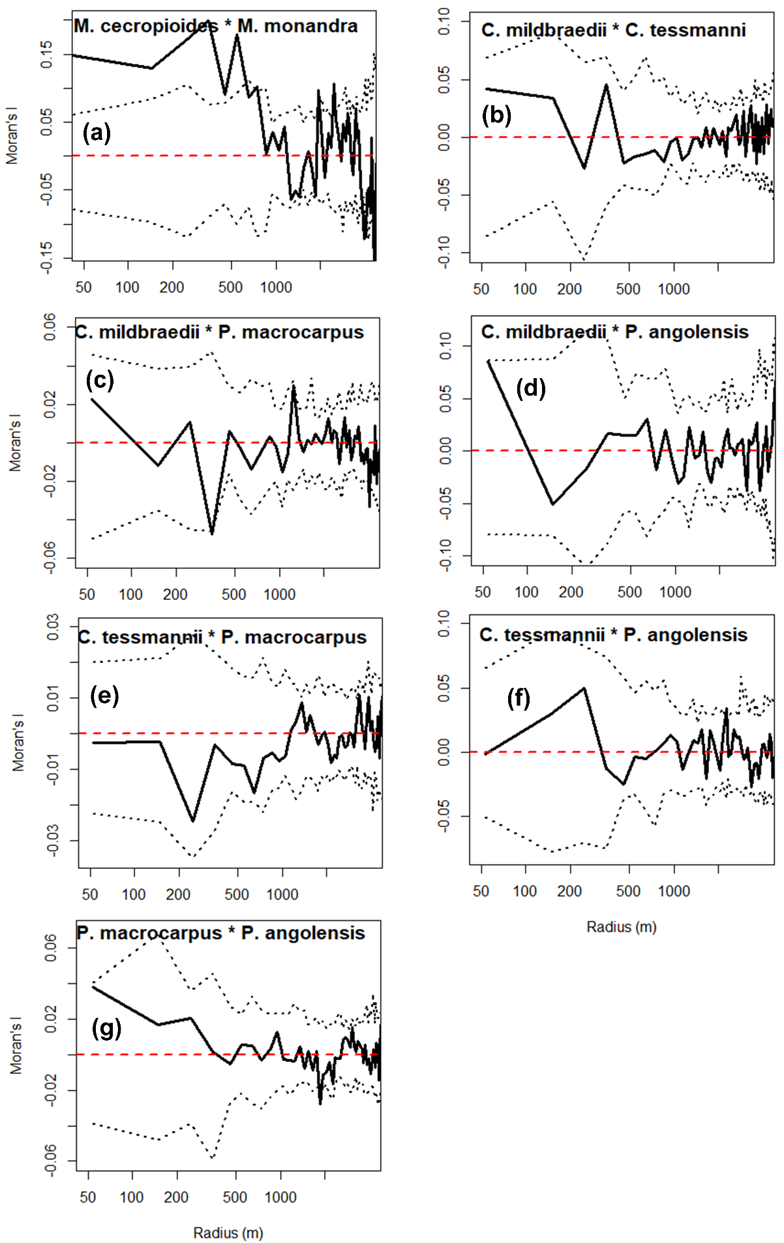

Supplement: Supplementary file 1 — Figure S1 [file ECE3-11-18691-s002.tif]
