# Supplementary material for: Spatial patterns of light‐demanding tree species in the Yangambi rainforest (Democratic Republic of Congo)
Source: Ecol Evol. 2021 Dec 20;11(24):18691–707. doi: 10.1002/ece3.8443 (PMC8717288; doi:10.1002/ece3.8443)
Supplement: Supplementary file 2 — Table S1‐S2 [file ECE3-11-18691-s001.docx]

**Appendix**

*Appendix 1. List of Study species and their ecological characteristics. Regeneration guild: SLP=short-lived pioneer, LLP=long-lived pioneer, NPLD=non-pioneer light-demanding and STS=shade-tolerant species. Dispersal mode: Au=autochory, An=anemochory, Z=zoochory, Hy=Hydrochory. Spatial distribution: G=gregarious, Ng=non-gregarious. Leaf phenology: D= deciduous, Sd=semi-deciduous, Nd=non-deciduous*

| **Guild** | **Taxa** | **Family** | **Fruit type** | **Diaspore morphology** | **Dispersal mode** | **Maximum DBH** | **Total height** | **Spatial distribution** | **Leaf phenology** |
| --- | --- | --- | --- | --- | --- | --- | --- | --- | --- |
| SLP | *Macaranga monandra* | Euphorbiaceae | Capsule | Seed | Au/Z |  | 25 m | G | Nd |
|  | *Macaranga spinosa* | Euphorbiaceae | Capsule | Seed | Au/Z |  | 20 m | G | Nd |
|  | *Macaranga zenkeri* | Euphorbiaceae | Capsule | Seed | Au/Z |  |  | G | Nd |
|  | *Musanga cecropioides* | Moraceae | Syncarpe | Achenes | Z | 80 cm | 30 m | G | Nd |
| LLP | *Albizia adianthifolia* | Fabaceae | Pod | Membranous pod | An | 50 cm | 25 m | Ng | D |
|  | *Albizia ferruginea* | Fabaceae | Pod | Membranous pod | An | 100 cm | 40 m | Ng | D |
|  | *Albizia gummifera* | Fabaceae | Pod | Membranous pod | An |  |  | Ng | D |
|  | *Albizia laurentii* | Fabaceae | Pod | Membranous pod | An |  |  | Ng | D |
|  | *Alstonia boonei* | Apocynaceae | Follicle | Small seed with long hairs | An | 150 cm | 40 m | Ng | D |
|  | *Canarium schweinfurthii* | Burseraceae | Drupe | Seed | Z | 200 cm | 50 m | Ng | D |
|  | *Ceiba pentandra* | Bombacaceae | Capsule | Small seeds with silky hairs | An/Hy | 200 cm | 50 m | Ng | D |
|  | *Erythrophleum suaveolens* | Caesalpiniaceae | Pod | Seed | Au/Z | 150 cm | 40 m | Ng | D |
|  | *Milicia excelsa* | Moraceae | Syncarpe | Achenes | Z | 150 cm | 55 m | Ng | D |
|  | *Nauclea diderrichii* | Rubiaceae | Fleshy infructescence | Seed | Z | 120 cm | 40 m | Ng | Nd |
|  | *Pericopsis elata* | Fabaceae | Pod | Flat pod | An | 160 cm | 50 m | G | Sd |
|  | *Piptadeniastrum africanum* | Mimosaceae | Pod | Winged seeds | An | 150 cm | 50 m | Ng | D |
|  | *Ricinodendron heudelotii* | Euphorbiaceae | Capsule | Seed | Z | 150 cm | 40 m | Ng | D |
| NPLD | *Afzelia bella* | Caesalpiniaceae | Pod | Seed | Au/Z | 140 cm | 40 m | Ng | D |
|  | *Afzelia bipindensis* | Caesalpiniaceae | Pod | Seed | Au/Z | 140 cm | 40 m | Ng | D |
|  | *Autranella congolensis* | Sapotaceae | Berry | Seed | Z | 200 cm | 50 m | Ng | D |
|  | *Celtis mildbraedii* | Ulmaceae | Drupe | Drupe | Z | 150 cm | 40 m | Ng | D |
|  | *Celtis tessmannii* | Ulmaceae | Drupe | Drupe | Z | 150 cm | 40 m | Ng | D |
|  | *Entandrophragma angolense* | Meliaceae | Capsule | Flattened and winged seeds | An | 150 cm | 50 m | Ng | D |
|  | *Entandrophragma candollei* | Meliaceae | Capsule | Flattened and winged seeds | An | 300 cm | 60 m | Ng | D |
|  | *Entandrophragma cylindricum* | Meliaceae | Capsule | Winged seeds | An | 200 cm | 60 m | Ng | D |
|  | *Entandrophragma utile* | Meliaceae | Capsule | Flattened and winged seeds | An | 200 cm | 60 m | Ng | D |
|  | *Khaya spp.* | Meliaceae | Capsule | Flattened and winged seeds | An | 150 cm | 60 m | Ng |  |
|  | *Lovoa trichilioides* | Meliaceae | Capsule | Winged seeds | An | 150 cm | 45 m | Ng | D |
|  | *Petersianthus macrocarpus* | Lecythidaceae | Samara | Samara with 4 wings | An | 150 cm | 30 m | Ng | D |
|  | *Pterocarpus soyauxii* | Fabaceae | Pod | Flattened and winged fruit | An | 150 cm | 50 m | Ng | D |
|  | *Pycnanthus angolensis* | Myristicaceae | Drupe | Ariled seed | Z | 150 cm | 35 m | Ng | Nd |
|  | *Pycnanthus marchalianus* | Myristicaceae | Drupe | Ariled seed | Z | 150 cm | 35 m | Ng | Nd |
| STS | *Gilbertiodendron dewevrei* | Caesalpiniaceae | Pod | Seed | Au | 200 cm | 40 m | G | Nd |

Source: (Whitmore et al., 1971; Prevost, 1983; Dalling et al., 1998; Tchiegang et al., 2001; De Madron et al., 2004; Thapliyal et al., 2005; Dick et al., 2007; Louppe et al., 2008; Zakaria et al., 2008; Lejoly et al., 2010; Boyemba, 2011; Bourland et al., 2012; Menga et al., 2012; Bourland et al., 2015; Abdullah et al., 2015; Meunier et al., 2015; Douh et al., 2018; Kasongo et al., 2018; Kafuti et al., 2020)

*Appendix 2. Number of individuals, trees density and DBH statistics for the inventoried species in the transects. Group of species: SLP=short-lived pioneer, LLP=long-lived pioneer, NPLD=non-pioneer light-demander and STS=shade-tolerant species. DBH: diameter at breast height. Sd: Standard deviation.*

| **Taxa** | **Individuals number** | **Relative percentage to total stems number** | **Tree density (stem ha-1)** | **DBH min** | **DBH mean** | **DBH max** | **DBH Sd** |
| --- | --- | --- | --- | --- | --- | --- | --- |
| **SLP guild** | **834** | **0.98%** | **3.328** |  |  |  |  |
| *Macaranga monandra* | 347 | 0.41% | 1.385 | 10.0 | 20.0 | 74.6 | 11.3 |
| *Macaranga spinosa* | 36 | 0.04% | 0.144 | 10.0 | 14.7 | 30.3 | 4.1 |
| *Macaranga zenkeri* | 9 | 0.01% | 0.036 | 10.6 | 15.2 | 20.1 | 4.1 |
| *Musanga cecropioides* | 442 | 0.52% | 1.764 | 10.0 | 30.9 | 78.7 | 13.9 |
| **LLP guild** | **800** | **0.94%** | **3.192** |  |  |  |  |
| *Albizia adianthifolia* | 45 | 0.05% | 0.180 | 12.4 | 35.4 | 86.9 | 18.4 |
| *Albizia ferruginea* | 12 | 0.01% | 0.048 | 22.8 | 63.7 | 87.6 | 18.6 |
| *Albizia gummifera* | 25 | 0.03% | 0.100 | 10.1 | 36.4 | 106.2 | 21.1 |
| *Albizia laurentii* | 1 | 0.00% | 0.004 | 13.9 | 13.9 | 13.9 | - |
| *Alstonia boonei* | 78 | 0.09% | 0.311 | 23.4 | 64.1 | 86.4 | 12.6 |
| *Canarium schweinfurthii* | 39 | 0.05% | 0.156 | 10.3 | 67.9 | 137.8 | 43.9 |
| *Ceiba pentandra* | 1 | 0.00% | 0.004 | 18.8 | 18.8 | 18.8 | - |
| *Erythrophleum suaveolens* | 295 | 0.35% | 1.177 | 10.3 | 55.9 | 125.0 | 27.2 |
| *Milicia excelsa* | 5 | 0.01% | 0.020 | 31.5 | 64.7 | 97.6 | 28.9 |
| *Nauclea diderrichii* | 23 | 0.03% | 0.092 | 14.4 | 64.7 | 105.7 | 32.4 |
| *Pericopsis elata* | 162 | 0.19% | 0.646 | 12.0 | 66.0 | 133.9 | 28.5 |
| *Piptadeniastrum africanum* | 82 | 0.10% | 0.327 | 10.1 | 45.5 | 146.1 | 31.1 |
| *Ricinodendron heudelotii* | 32 | 0.04% | 0.128 | 10.0 | 30.5 | 86.0 | 18.3 |
| **NPLD guild** | **4694** | **5.54%** | **18.729** |  |  |  |  |
| *Afzelia bella* | 9 | 0.01% | 0.036 | 10.8 | 22.0 | 61.0 | 16.7 |
| *Afzelia bipindensis* | 5 | 0.01% | 0.020 | 17.7 | 28.1 | 36.0 | 8.1 |
| *Autranella congolensis* | 24 | 0.03% | 0.096 | 10.4 | 29.0 | 131.2 | 31.6 |
| *Celtis mildbraedii* | 350 | 0.41% | 1.397 | 10.0 | 25.9 | 121.2 | 21.3 |
| *Celtis tessmannii* | 860 | 1.02% | 3.431 | 10.0 | 26.3 | 87.6 | 14.4 |
| *Entandrophragma angolense* | 106 | 0.13% | 0.423 | 10.1 | 34.7 | 133.4 | 28.7 |
| *Entandrophragma candollei* | 71 | 0.08% | 0.283 | 10.0 | 28.8 | 166.0 | 36.4 |
| *Entandrophragma cylindricum* | 46 | 0.05% | 0.184 | 11.6 | 50.1 | 182.0 | 48.8 |
| *Entandrophragma utile* | 61 | 0.07% | 0.243 | 10.0 | 25.9 | 110.0 | 23.3 |
| *Lovoa trichilioides* | 22 | 0.03% | 0.088 | 10.4 | 28.8 | 91.3 | 23.6 |
| *Petersianthus macrocarpus* | 2407 | 2.84% | 9.604 | 10.0 | 27.2 | 99.1 | 15.0 |
| *Pterocarpus soyauxii* | 344 | 0.41% | 1.373 | 10.0 | 49.6 | 120.0 | 26.7 |
| *Pycnanthus angolensis* | 388 | 0.46% | 1.548 | 10.0 | 16.9 | 98.7 | 10.6 |
| *Pycnanthus marchalianus* | 1 | 0.00% | 0.004 | 33.7 | 33.7 | 33.7 | - |
| **STS guild** | **1148** | **1.36%** | **4.581** |  |  |  |  |
| *Gilbertiodendron dewevrei* | 1148 | 1.36% | 4.581 | 10.0 | 43.5 | 144.7 | 26.2 |
